# Neuroprotection after cardiac arrest with 2-iminobiotin: a single center phase IIa study on safety, tolerability, and pharmacokinetics

**DOI:** 10.3389/fneur.2023.1136046

**Published:** 2023-06-02

**Authors:** M. M. Admiraal, D. C. Velseboer, H. Tjabbes, P. Vis, C. Peeters-Scholte, J. Horn

**Affiliations:** ^1^Department of Clinical Neurophysiology, Amsterdam UMC, Amsterdam, Netherlands; ^2^Amsterdam Neurosciences, Amsterdam, Netherlands; ^3^Department of Intensive Care, Amsterdam UMC, Amsterdam, Netherlands; ^4^Neurophyxia BV, ’s-Hertogenbosch, Netherlands; ^5^LAP&P Consultants BV, Leiden, Netherlands

**Keywords:** cardiac arrest, neuroprotection, nitric oxide synthase, 2-iminobiotin, pharmacokinetics

## Abstract

**Background:**

Brain injury is a serious problem in patients who survive out-of-hospital cardiac arrest (OHCA). Neuroprotective drugs could reduce hypoxic–ischemic reperfusion injury. The aim of this study was to investigate the safety, tolerability, and pharmacokinetics (PK) of 2-iminobiotin (2-IB), a selective inhibitor of neuronal nitric oxide synthase.

**Methods:**

Single-center, open-label dose-escalation study in adult OHCA patients, investigating three 2-IB dosing schedules (targeting an AUC_0-24h_ of 600–1,200 ng*h/m in cohort A, of 2,100–3,300 ng*h/mL in cohort B, and 7,200–8,400 of ng*h/mL in cohort C). Safety was investigated by monitoring vital signs until 15 min after study drug administration and adverse events up to 30 days after admission. Blood sampling for PK analysis was performed. Brain biomarkers and patient outcomes were collected 30 days after OHCA.

**Results:**

A total of 21 patients was included, eight in cohort A and B and five in cohort C. No changes in vital signs were observed, and no adverse events related to 2-IB were reported. A two-compartment PK model described data the best. Exposure in group A (dosed on bodyweight) was three times higher than targeted (median AUC_0-24h_ 2,398 ng*h/mL). Renal function was an important covariate; therefore, in cohort B, dosing was performed on eGFR on admission. In cohort B and C, the targeted exposure was met (median AUC_0-24h_ 2,917 and 7,323 ng*h/mL, respectively).

**Conclusion:**

The administration of 2-IB to adults after OHCA is feasible and safe. PK can be well predicted with correction for renal function on admission. Efficacy studies with 2-IB after OHCA are needed.

## Introduction

Out-of-hospital cardiac arrest (OHCA) remains a major cause of death despite improvements such as rapid response teams and the use of automatic external defibrillators. When cardiopulmonary resuscitation is successful, patients are admitted to the intensive care unit (ICU), where the major aim of treatment is to minimize hypoxic–ischemic reperfusion injury. The most recent guidelines advice target temperature management (TTM) besides optimal supportive ICU care (1). Despite this therapy, 50% of the patients admitted to the ICU after OHCA die, mainly due to severe brain injury (2). Theoretically, brain injury could be diminished by neuroprotective agents interfering with the complex cascade of reperfusion injury, including neuronal excitotoxicity, oxidative stress, mitochondrial dysfunction, and disrupted calcium homeostasis, leading to apoptosis- and necrosis-mediated cell death (3).

Many promising neuroprotective agents were investigated in large trials with ischemic stroke patients in the late 1990s. Studies were negative, showing no beneficial effects or serious side effects (4). However, these studies were done in patients with occluded cerebral arteries leading to stroke. In OHCA patients, all cerebral arteries are patent, so a neuroprotective drug can easily reach the target cells after the return of spontaneous circulation. Several neuroprotective agents, such as exenatide, magnesium, and erythropoetin, have been investigated in OHCA, but a proven effective drug is currently not available (5).

Nitric oxide (NO) plays a critical role in the development of reperfusion injury. After hypoxia-ischemia, it is synthesized through the action of constitutive present nitric oxide synthases (NOS) isoenzymes, i.e., the neuronal and endothelial NOS and the upregulation of inducible NOS. Selectively blocking the harmful neuronal and inducible NOS with 2-iminobiotin (2-IB), a biotin (Vitamin H or B7) analog, might be an effective strategy in the treatment of reperfusion injury (6). Animal model studies with this agent showed promising results. Furthermore, in clinical studies in neonates after hypoxia-ischemia due to perinatal asphyxia, a type of brain injury comparable to OHCA patients, administration of 2-IB was feasible and safe (7, 8).

In this study, we administered 2-IB, a selective inhibitor of neuronal and inducible NOS, to OHCA patients. The aim of this study was to investigate the safety, tolerability, and pharmacokinetics (PK) of 2-IB in this population.

## Materials and methods

### Design

This was an investigator-initiated, unblinded, single-center prospective cohort study. A total of three cohorts (*n* = 8) with different dosing schedules were planned. The study protocol was approved by the ethical committee of the Amsterdam Medical Center (NL 54915, EUDRACT number 2015–003902-17) and registered at: NCT02836340. The study was conducted in adherence to (inter) national standards of good clinical practice and was monitored by the Clinical Research Centre of the Amsterdam University Medical Centers.

After the completion of each cohort, inclusion was temporarily stopped. The results were analyzed and discussed with the Data and Safety Monitoring Safety Board (DSMB). If necessary, the study protocol was adapted and submitted to the ethical committee for review and approval.

### Participants

Consecutive adult patients admitted to the ICU after OHCA were screened for eligibility by the physician on call. If the patients fulfilled the inclusion criteria, one of the investigators (MMA and JH) came to the hospital for inclusion and informed consent. Inclusion criteria for cohort A were as follows: (1) OHCA due to a cardiac cause, (2) coma on ICU admission, (3) treatment with TTM at 36°C, and (4) possibility of administration of study medication within 6 h after OHCA via a central venous catheter. After analyzing the results of cohort A, two criteria were added for cohort B and C: (1) duration of resuscitation not longer than 30 min and (2) shockable rhythm as presenting rhythm. Administration via a central venous catheter was not required anymore. Instead, using a peripheral intravenous catheter was allowed based on additional safety data from other trials (7, 8). Exclusion criteria in all cohorts were as follows: (1) female patient aged < 50 years, (2) known co-morbidity with a life expectancy of less than 6 months prior to OHCA, (3) known severe cognitive impairment prior to OHCA, and (4) no informed consent.

As all patients were unconscious on ICU admission, a legal representative was informed about the study and asked for informed consent before the start of study medication. If the patient recovered, informed consent for the use of collected data and follow-up was asked.

### Intervention and ICU care

All patients received intravenously administered 2-IB (Neurophyxia BV, ‘s Hertogenbosch, Netherlands). Preferably, a central venous catheter was used for administration, but if this was not available, administration using a peripheral intravenous catheter was allowed. Each patient received a total of six dosages of study medication in 24 h (every 4 h), infused in 15 min.

A total of three cohorts of patients were included using a dose-escalation design: In cohort A, cohort B, and cohort C, an exposure was targeted at an AUC_0-24h_ of 600–1,200 ng*h/mL, 2,100–3,300 ng*h/mL, and 7,200–8,400 ng*h/Ml, respectively. Based on preclinical data and human neonatal pharmacokinetics (PK) data, a dose of 0.055 mg/kg/dose was administered in cohort A (8). After completion of cohort A, exposure was found to be higher than expected and renal function turned out to be an important co-variate in the determination of this exposure. Therefore, in cohort B and C, dosing was based on renal function, which was determined using the estimated glomerular filtration rate (eGFR) on hospital admission (see Supplementary Table S1 for the dosing schedule). The eGFR was calculated using the Modification of Diet in Renal Disease (MDRD) equation: 186 × (Creat/88.4)^–1.154^ × (Age)^–0.203^ × (0.742 if female) × (1.210 if the patient is black) (9). The MDRD is the clinical standard in Netherlands to calculate eGFR.

Standard ICU care for OHCA patients consisted of TTM with a target of 36°C for 24 h during which patients were sedated using propofol and, if needed, remifentanil. Mechanical ventilation, hemodynamic monitoring, and, if necessary, vasopressive and inotropic support, were applied in all patients. After completion of TTM, sedative medication was stopped, and the neurological situation was evaluated. If the patient remained comatose, the neurologist was consulted for advice on prognostication. The neurologist was unaware of the participation in the current study and national guidelines for prognostication were used.

### Endpoints

The primary objective was to explore the short-term safety, tolerability, and PK of 2-IB. The main study parameters used for evaluating the short-term safety and tolerability were (1) vital signs, i.e., heart rate, blood pressure, ST segment changes as sign of cardiac ischemia, assessed every 5 min before and until 15 min after 2-IB administration, (2) biochemistry and hematology measured as part of clinical care, (3) the occurrence of serious adverse events (SAEs) up to 7 days in the ICU or until ICU discharge, whichever occurred first, and (4) adverse events (AE) up to 30 days.

For outcome assessment, the Cerebral Performance Category (CPC) scale was used (10). The best CPC score in 30 days after OHCA was registered. Poor outcome was defined as a CPC score of 3–5 [severely disabled, vegetative state, and (brain) death].

Brain injury was assessed using the neurobiomarker neuron specific enolase (NSE), S100ß and neurofilament light chain (NfL) at 24 h and 48 h (± 1 h) after the first dose of study medication (11–13).

Other data collected include demographic and baseline characteristics, OHCA details, cardiac arrest hospital prognosis (CAHP) score, hospital data (laboratory results, neurological examination after clearance of sedative drugs, and length of stay (LOS) in the ICU and in the hospital) (14).

For evaluation of PK of 2-IB, nine 3.5 mL plasma samples were taken from an arterial catheter at 1 min after the end of the first administration, 45 min and 1 h 15 min after the start of the first administration and just before the start of the second administration, 1 min before the 4th, 5th, or 6th administration, 1 min after the end of this administration and 45 min, and 1 h 15 min and 4 h after the start of the chosen administration. Except for the first blood sample, in all these time points, a margin of ± 5 min was used. Blood samples were centrifuged, and the supernatant was kept frozen at − 80°C until determination. The following PK parameters were determined: observed maximum plasma concentration (C_max_), area under the plasma concentration–time curve from time 0–24 h after administration (AUC_0–24h_), time at maximum plasma concentration (*T*_max_), terminal elimination half-life (*t*_1/2_), clearance (CL), and volume of distribution (Vd).

### Pharmacokinetics population model

Pharmacokinetics analysis was performed using nonlinear mixed effects modeling (NONMEM; version 7.4; ICON Development Solutions, Ellicott City, MD, United States), with the main aim to obtain individual exposure metrics for 2-IB (15). With the data from cohort A, an initial population PK model was built. The estimation method was the first-order conditional estimation with an interaction option in NONMEM. Simulation studies were performed to identify the best dose for use in cohort B with dose adjustment for renal function at admission. Using the eGFR on admission, a dosing schedule for cohort B was defined. In order to reach the target of cohort C, a three times higher dose compared to cohort B was given in cohort C based on eGFR. The population PK model was reevaluated with data from cohort A and B after cohort B was completed, and again with all data after completion of cohort C.

### Analyses of clinical characteristics

Baseline characteristics, distributions on the CPC, and outcome measures are presented in a descriptive way as mean ± SEM or as percentages when appropriate. Not normally distributed data were reported as median [with interquartile range (IQR)].

## Results

**Figure 1:**
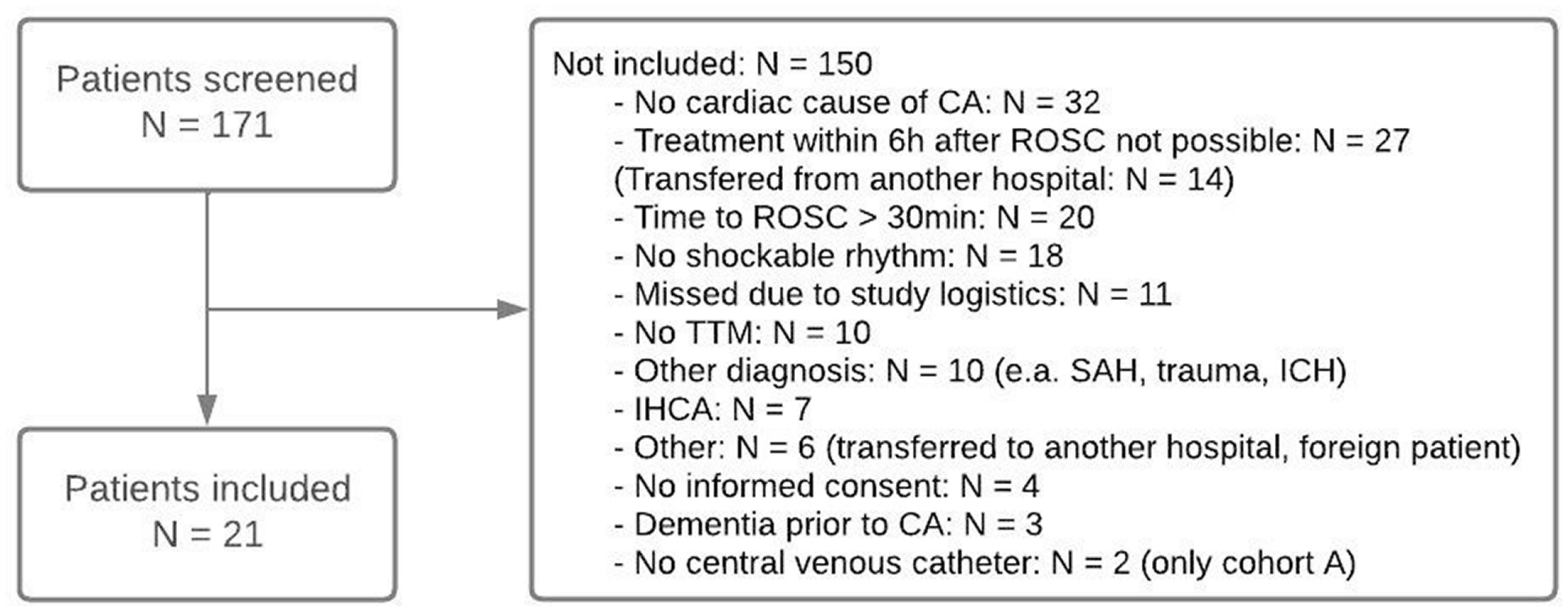
Flowchart of patient inclusions.

Between May and October 2016, the first eight patients (cohort A) were included in the study, after which inclusion was stopped to enable the analysis of the results. After DSMB approval and protocol amendment based on the findings in cohort A, inclusion was restarted in May 2017 and finished in December 2017 when the next eight patients were included (cohort B). One patient, patient 12, should not have been included because the inclusion criterion of cohort B (no shockable rhythm and a resuscitation time exceeding 30 min) was not fulfilled. After analysis and approval of the DSMB, the inclusion of cohort C was started in January 2019 and finished in February 2020. Because inclusion rate was low and with the start of the emerging COVID-19 pandemic, we decided to stop inclusion after five patients. In total, 171 patients were screened during the periods of inclusion. Reasons for exclusion are shown in the flow chart (Figure 1).

### Patient data

Baseline characteristics, OHCA details, and outcome of the included patients are presented in Table 1. The duration of resuscitation was longer, and CAHP scores were higher in cohort A compared with cohort B and C, which was caused by a change in inclusion criteria. Study medication was started at a median of 5.3 h (IQR 4.8–5.6) after OHCA. In 20 patients, all six dosages were administered as scheduled. One patient (patient 18) died due to refractory cardiogenic shock with multiorgan failure before the last dosage was administered.

**Table 1:** Baseline characteristics and outcome.

	Cohort A (*n* = 8)	Cohort B (*n* = 8)	Cohort C (*n* = 5)
Male n	7	5	4
Age, median [IQR]	60.5 [59.5–67]	64 [63–66]	74 [69–76]
Body weight, median [IQR]	87.5 [82–112]	76.5 [67.5–96.5]	83 [77–84]
Witnessed arrest, n	8	7	5
Time to ROSC, minutes median [IQR]	17.5 [7.5–48]	10 [9–10], 1 unknown	21 [15–25]
Initial rhythm VF/VT, n	8	6, 1 unknown	5
CAHP score, median [IQR]	0.86 [0.23–0.97]	0.28 [0.22–0.53]	0.50 [0.45–0.89]
LOS ICU, days, median [IQR]	2.5 [2–3]	3 [2.5–3]	2 [2–2.5]
LOS hospital, days, median [IQR]	3 [2–4]	8 [3.5–15]	3 [3–3]
CPC score, median [IQR]	3.5 [1.5–5]	1 [1–2]	4 [2–4]
Poor outcome (CPC score 3–5), n	4	1	3
Mortality, n	4	1	3

CAHP, cardiac arrest hospital prognosis; ICU, intensive care unit; IQR, interquartile range; LOS, length of stay; ROSC, return of spontaneous circulation; VF, ventricular fibrillation; VT, ventricular tachycardia.

### Safety and tolerability

**Figure 2:**
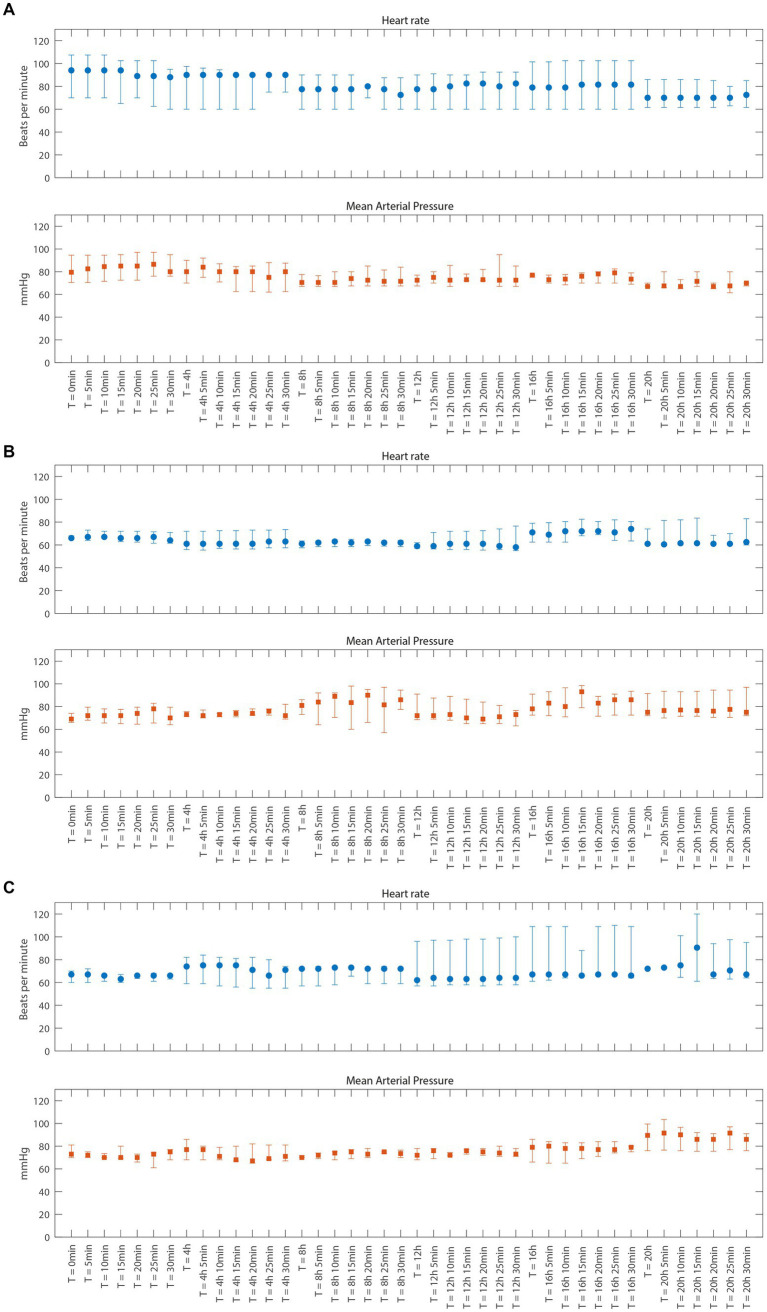
Vital parameters just before and 5 through 30 min after each dose of 2-Immunobiotin for cohort A, B, and C **(A–C)**, respectively. Data are presented as median and interquartile range. **(A)** Cohort A **(B)** Cohort B **(C)** Cohort C.

In all patients, the study medication was well tolerated, and no safety concerns were encountered during the 24 h of administration of 2-IB. No statistically significant or clinically relevant changes in heart rate or blood pressure were observed up to 15 min after 2-IB administration compared to before administration in either cohort (see Figures 2A–C). No signs of cardiac ischemia (ST segment changes) were seen during the 24 h administration period of the study medication in either cohort. The laboratory results of standard blood tests did not show any unexpected deviations for this patient population.

In total, eight patients died (four in cohort A, one in cohort B, and three in cohort C), which was reported as an SAE to the DSMB and regulation authorities. In all cases, the SAE was judged as not related to the study medication. One patient in cohort A suffered another cardiopulmonary arrest, consisting of bradycardia with loss of circulation. This patient was still in the ICU, and CPR was performed immediately and successfully. This SAE occurred more than 24 h after the last administration of the study drug. In cohort B, one patient woke up very agitated after sedative drugs were stopped. It was decided to extubate the patient to see if this affected the agitation. As no improvement occurred, sedative medication had to be restarted which necessitated reintubation and mechanical ventilation for another 24 h. Finally, in cohort C, one patient was readmitted to the hospital after discharge because of shortness of breath and cystitis with urinary retention. This occurred within 30 days after inclusion in the study and was scored as an SAE. After treatment with diuretics and antibiotics, the patient recovered rapidly. These three SAEs were also judged as not related to the study medication.

### Secondary outcomes

**Figure 3:**
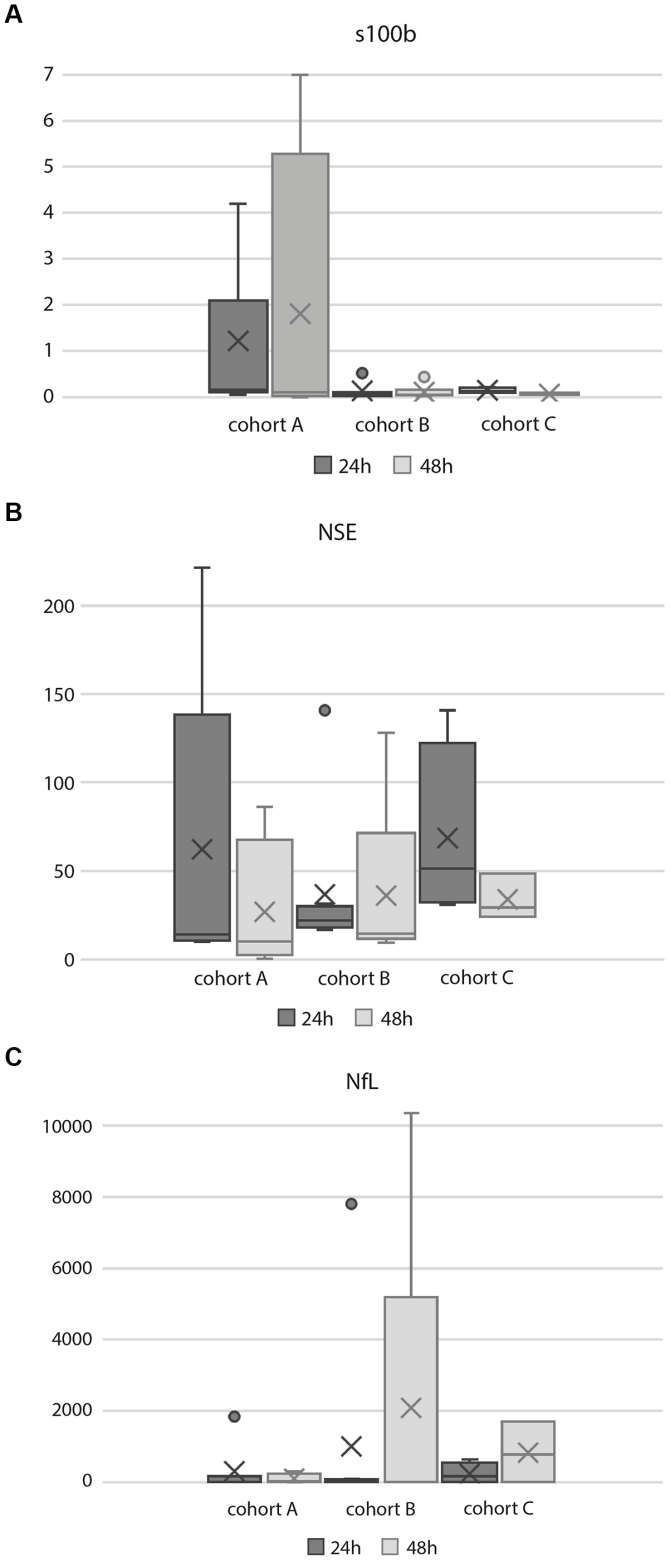
Box-whisker plots of brain injury biomarkers. **(A)** s100b, **(B)** NSE, **(C)** NfL.

A total of eight patients died, seven due to brain injury leading to postanoxic coma and a decision to withdraw life-sustaining therapy (Table 1). One patient died due to cardiogenic shock. Brain injury markers are presented in Table 2 and Figure 3.

**Table 2:** Results of brain injury markers.

	Cohort A (*n* = 8)	Cohort B (*n* = 8)	Cohort C (*n* = 5)
S100b 24 h	0.2 [0.1–2.0] *N* = 7	0.1 [0.0–0.1]	0.1 [0.1–0.2] *N* = 4
S100b 48 h	0.1 [0.1–1.8] *N* = 4	0.1 [0.0–0.1] *N* = 5	0.1 [0.1–0.1] *N* = 3
NSE 24 h	14.1 [11.3–55.1] *N* = 5	21.8 [18.1–28.0]	51.2 [34.9–84.8] *N* = 4
NSE 48 h	10.1 [6.8–30.0] *N* = 4	14.5 [13.9–15.0] *N* = 5	29.4 [26.7–38.9] *N* = 3
NfL 24 h	20.5 [14.7–111.9] *N* = 7	30.1 [10.9–49.6]	162.1 [12.8–392.6] *N* = 4
NfL 48 h	35.4 [19.2–112.2] *N* = 4	18.6 [12.3–27.9] *N* = 5	778.6 [397.7–1241.9] *N* = 3

CPC, cerebral performance category; NfL, neurofilament light; NSE, neuron specific enolase.

### Pharmacokinetics

In all samples of cohort A, B, and C, 2-IB concentrations could be measured; no concentrations were below the lower limit of quantification. The median value of clearance was 12.3 L/h, volume of distribution was 10 L, and half-life was 1.5 h. Individual values can be found in Supplementary Table S2.

#### Cohort A

The median AUC_0–24h_ for cohort A was 2,398 ng*h/mL (range 1,606–6,111 ng*h/mL), nearly three times the target AUC_0–24h_ of 600–1,200 ng*h/mL (see Supplementary Table S2 for individual data). For individual PK fits of patients 1–8, see Supplementary Figure S1, Panel A. Based on these results, a population PK model was developed. A two-compartment structural PK model was superior to a one-compartment model and provided an adequate fit for the data.

#### Cohort B

The median AUC_0–24h_ for cohort B was 2,479 ng*h/mL (ranging from 2,102 to 4,737 ng*h/mL), nearly equaling the target AUC_0–24h_ of 2,100–3,300 ng*h/mL (see Supplementary Table S2). Individual PK fits are shown in Supplementary Figure S1, Panel B. Analysis of PK data of cohort B showed that the 2-compartment PK model, with a dependence of clearance on eGFR, still held. In order to reach a target AUC_0–24h_ of 7,200–8,400 ng*h/mL, a three times higher dose compared to cohort B was given in cohort C based on eGFR.

#### Cohort C

After the inclusion of five patients in cohort C, the PK model still held the same covariate influence of eGFR on clearance. The median AUC_0–24h_ for cohort C was 7,323 ng*h/mL (ranging from 5,641 to 27,866 ng*h/mL), reaching the target AUC_0–4h_ of 7,200–8,400 ng*h/mL (see Supplementary Table S2). Individual PK fits are shown in Supplementary Figure S1, panel C.

For more PK details, see Supplementary Table S3.

## Discussion

The results of this open-label, dose-escalating phase 2a study suggest that 2-IB can be safely administered to patients admitted to the ICU after OHCA. No serious problems regarding vital signs were encountered, even in the high-range dosage schedule of the last cohort. Dosing on body weight did not yield a predictable exposure, but after adjustment for renal function, as measured with the eGFR on admission, a stable prediction of 2-IB exposure was possible.

Despite treatment in the ICU with TTM, mortality in studies including OHCA patients is nearly 50%. The main cause of death is severe post-hypoxic–ischemic brain injury (2). Reperfusion sets off a cascade of biochemical changes that result in this brain injury. Many different neuroprotective drugs have been developed and tested for this indication, but none showed unequivocal benefit (5). It is uncertain whether one drug, affecting only one step in this complicated process, will be the final solution (3). In our study, the outcome (CPC and mortality) was the best in cohort B. However, due to the small sample size, no inferences can be drawn.

Optimal dosing of new drugs is important to achieve optimal effectiveness but also avoid too high, possibly harmful levels. In all cohorts in the current study, no (severe) adverse events were found that could be contributed to the use of 2-IB. In the first eight patients, body weight was used to define the dosing schedule. PK showed that very high levels were reached, with renal function being a major covariate. Therefore, cohort B and C received medication based on eGFR for which admission data were used. This yielded satisfying PK data. In patients with multiorgan failure, kidney function often deteriorates during the first days of admission. As treatment was only given for 24 h, no adaptation of the study drug was necessary. But when long-term administration is considered, the adaptation of dosing would be necessary. In this study, 2-IB was infused in 15 min every 4 h, the same schedule as in preclinical trials (16, 17). In later safety and PK studies, in neonates, it was shown that a bolus administration in 1–2 min was well tolerated and did not lead to a drop in blood pressure (7, 8).

Some limitations of the study need to be discussed. The study was a relatively small single-center project, with only a limited number of patients in each cohort, especially in the last cohort. We found that inclusion of patients was hampered as this was done in the ICU after achieving informed consent from relatives. Family members were often unavailable shortly after admission, or time was running out before informed consent could be achieved. For optimal conduction of these types of acute intervention studies, deferred consent is needed (18). This will also allow early administration of study medications, enabling research on optimal effectiveness. Ideally, neuroprotective drugs are administered as soon as possible after return of circulation, rather than even before hospital admission.

Despite these limitations, the results show that 2-IB can be administered to adult patients after OHCA safely. The optimal dosing should be based on kidney function on admission. Further studies are needed to investigate the effectiveness.

Furthermore, selection of patients most likely to benefit from such a treatment is needed. Patients admitted after a very long resuscitation are very likely to have a poor outcome despite optimal treatment (19). To specifically target patients with a potential benefit of the treatment, we changed the inclusion criteria after inclusion of the first eight patients. On the contrary, patients who survive a cardiac arrest with a short resuscitation, usually make a good recovery. These patients are also unlikely to benefit from additional neuroprotective drugs. For a phase III trial, we would suggest to include OHCA patients with a reasonable chance of survival but with neurological sequelae. Tools to identify such patients on admission, for instance, the CAHP score, could be useful (14).

## Conclusion

Administration of 2-IB to patients admitted after OHCA is feasible and safe. PK is predictable when dosing is corrected for renal function on admission. For future studies on efficacy, deferred consent procedures are needed to allow administration as soon as possible. Furthermore, optimal patient selection is needed to include patients with a reasonable chance of survival.

## Data availability statement

The raw data supporting the conclusions of this article will be made available by the authors, without undue reservation.

## Ethics statement

The studies involving human participants were reviewed and approved by Amsterdam Medical Center. The patients/participants provided their written informed consent to participate in this study.

## Author contributions

MA, CP-S, and JH: conception and design, analysis and interpretation of data, drafting the manuscript. MA and JH conducted the study, selected patients, collected data, and performed the safety analysis. PV performed the pharmacokinetic analysis and advised on the 2-IB dose used for this study. CP-S and HT advised on the study protocol and supplied several other documents required for ethics committee approval. JH drafted the manuscript. The other authors provided critical revision of the intellectual content. All authors contributed to the article and approved the submitted version.

## Conflict of interest

CP-S is one of the inventors of 2-iminobiotin as a neuroprotective agent for cerebral hypoxia ischemia and/or reperfusion injury. CP-S and HT are shareholders and consultants of Neurophyxia BV. PV is employed at LAP&P Consultants BV.

The remaining authors declare that the research was conducted in the absence of any commercial or financial relationships that could be construed as a potential conflict of interest.

## Publisher’s note

All claims expressed in this article are solely those of the authors and do not necessarily represent those of their affiliated organizations, or those of the publisher, the editors and the reviewers. Any product that may be evaluated in this article, or claim that may be made by its manufacturer, is not guaranteed or endorsed by the publisher.
