# Supplementary material for: Neuroprotection after cardiac arrest with 2-iminobiotin: a single center phase IIa study on safety, tolerability, and pharmacokinetics
Source: Front Neurol. 2023 Jun 2;14:1136046. doi: 10.3389/fneur.2023.1136046 (PMC10272808; doi:10.3389/fneur.2023.1136046)
Supplement: Supplementary file 1 [file Data_Sheet_1.docx]

Table S1: dosing scheme

| Weight | Cohort A | eGFR | Cohort B | Cohort C |
| --- | --- | --- | --- | --- |
| kg | mg | ml/min/1.73m^2^ | mg | mg |
| 45-49 | 2.48 | 0-29 | 3.0 | 9.0 |
| 50-54 | 2.75 | 30-39 | 3.8 | 11.4 |
| 55-59 | 3.03 | 40-49 | 4.5 | 13.5 |
| 60-64 | 3.30 | 50-59 | 6.0 | 18.0 |
| 65-69 | 3.58 | 60-79 | 6.8 | 20.4 |
| 70-74 | 3.85 | 80-99 | 7.5 | 22.5 |
| 75-79 | 4.13 | 100-124 | 8.3 | 24.9 |
| 80-84 | 4.40 | 125-149 | 9.8 | 29.4 |
| 85-89 | 4.68 | 150-174 | 11.3 | 33.9 |
| 90-94 | 4.95 | 175-199 | 12.0 | 36.0 |
| 95-99 | 5.23 | 200-220 | 12.8 | 38.4 |
| 100-104 | 5.50 |  |  |  |
| 105-109 | 5.78 |  |  |  |
| 110-114 | 6.05 |  |  |  |
| 115-119 | 6.33 |  |  |  |
| 120-124 | 6.60 |  |  |  |
| ≥ 125 | 6.88 |  |  |  |

Dosing was based on eGFR (in ml/min/1.73m^2^) on hospital admission.

Table 2S: Individual PK metrics

| ID | Cmax | Tmax | T½ | AUC_0-24h_ |
| --- | --- | --- | --- | --- |
|  | (ng/mL) | (h) | (h) | (ng.h/mL) |
| Cohort A |  |  |  |  |
| 1 | 446 | 16.2 | 1.5 | 2052 |
| 2 | 411 | 20.22 | 1.2 | 2085 |
| 3 | 207 | 8.2 | 1.6 | 1700 |
| 4 | 519 | 8.12 | 1.1 | 1606 |
| 5 | 501 | 16.33 | 4 | 6111 |
| 6 | 499 | 20.25 | 3 | 4952 |
| 7 | 517 | 8.02 | 1.6 | 2711 |
| 8 | 376 | 20.17 | 5.6 | 5123 |
| Median | 473 | 16 | 1.6 | 2398 |
|  |  |  |  |  |
| Cohort B |  |  |  |  |
| 9 | 594 | 16.23 | 1.1 | 2610 |
| 10 | 511 | 16.25 | 1.8 | 3769 |
| 11 | 819 | 16.27 | 1.9 | 4737 |
| 13 | 732 | 0.17 | 1.1 | 2479 |
| 14 | 616 | 12.05 | 1.2 | 2440 |
| 15 | 445 | 20.25 | 1.4 | 2285 |
| 16 | 565 | 4.09 | 1.1 | 2102 |
| Median | 594 | 16.23 | 1.2 | 2479 |
|  |  |  |  |  |
| Cohort C |  |  |  |  |
| 17 | 1774 | 16.18 | 1.4 | 7323 |
| 18 | 2552 | 16.23 | 7.1 | 27866 |
| 19 | 1436 | 16.18 | 1 | 5641 |
| 20 | 1272 | 8.26 | 1.5 | 8545 |
| 21 | 1420 | 12.25 | 1.1 | 6386 |
| Median | 1436 | 16.18 | 1.4 | 7323 |

Patient 12 is not reported since it did not fulfill the inclusion criteria.

Abbreviations: Cmax: maximum plasma concentration, Tmax: time after first dose at which maximum plasma concentration is reached, T1/2 elimination half-life, AUC4h area under the concentration time curve based on PK parameter estimates from group A-C.

Figure 1S: Individual PK fits per cohort

A.


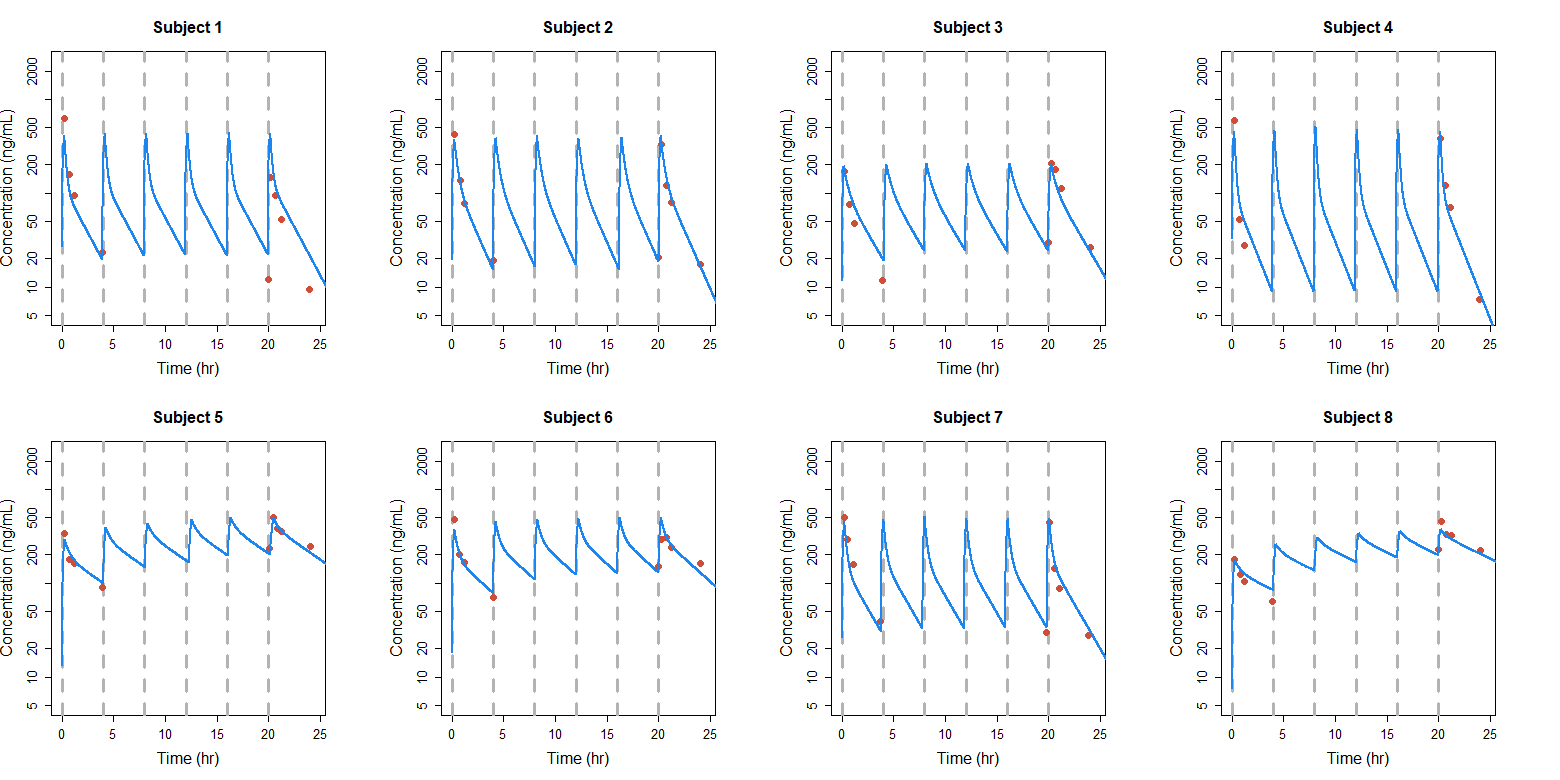


B.


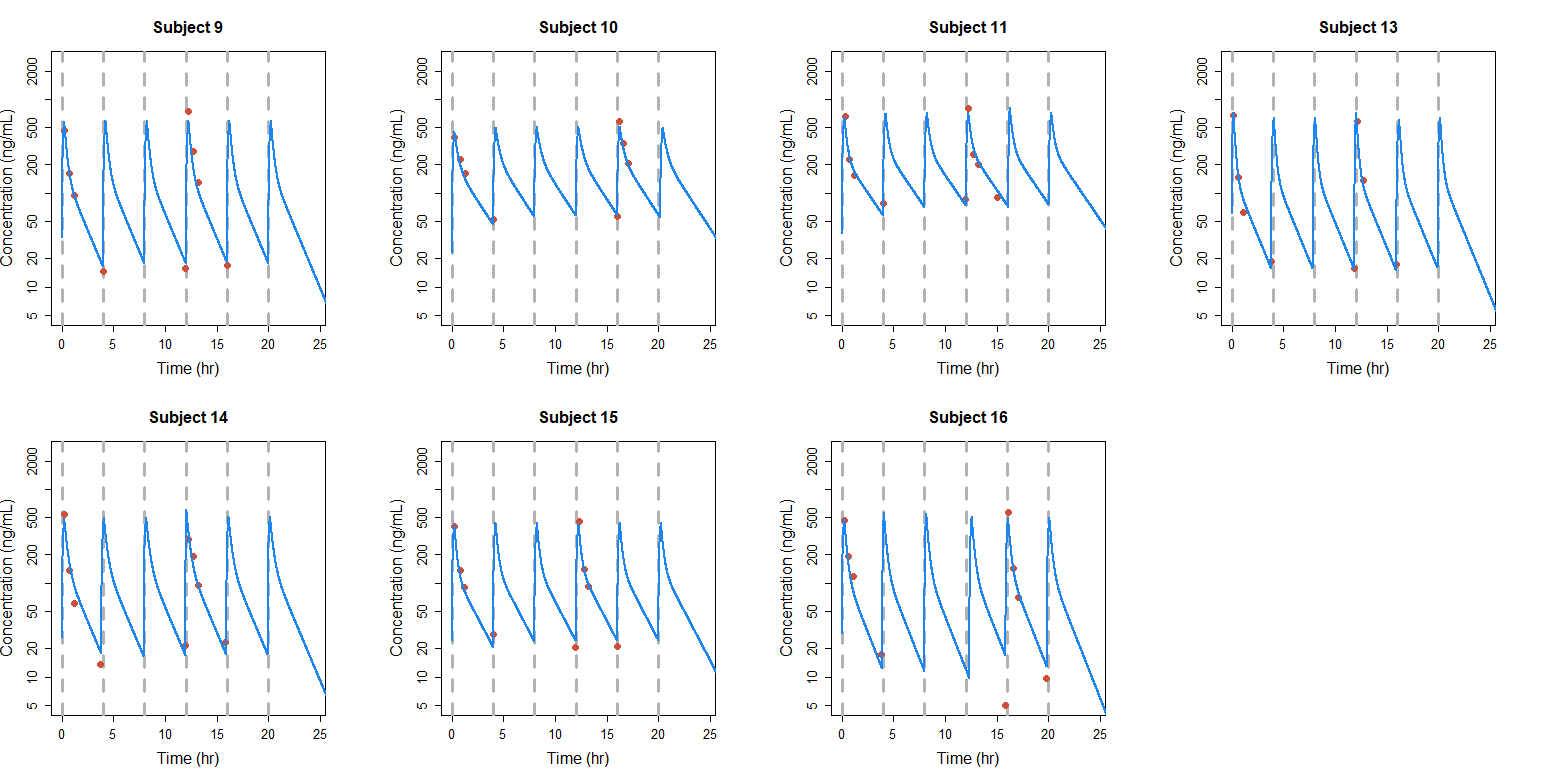


C.


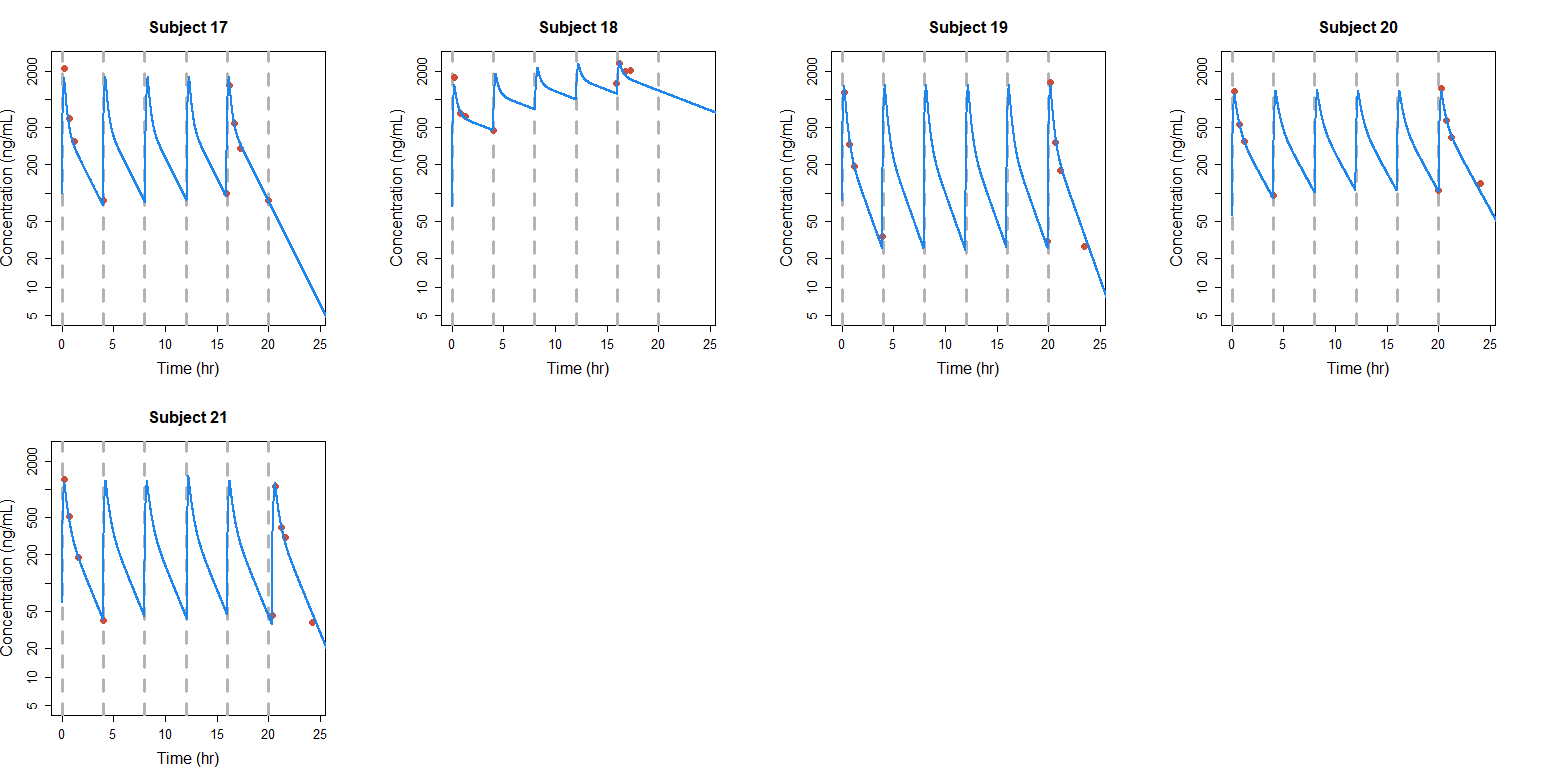


Table 3S: Population pharmacokinetic parameters for 2-IB in adults after out-of-hospital cardiac arrest based on data from cohorts A, B and C (n-21)

| Parameter | Estimate (SE) | CV (%) | 95% CI |
| --- | --- | --- | --- |
| Clearance (L/h) | 12.10 (1.31) | 12 | 9.55-14.70 |
| eGFR0 on Clearance | 1.03 (0.43) | 42 | 0.19-1.87 |
| Vcentral (L) | 10.1 (1.17) | 12 | 7.77-12.30 |
| Q (L/h) | 14.6 (1.15) | 7.9 | 12.3-16.80 |
| Vperipheral (L) | 10.8 (0.72) | 6.7 | 9.34-12.20 |
|  |  |  |  |
| Inter-individual variability | 0.225 (0.09) | 41 | 0.04-0.41 |
| IIV Vcentral | 0.27 (0.10) | 36 | 0.08-0.47 |
| Residual Error | 0.06 (0.02) | 28 | 0.03-0.10 |

Abbreviations: CI confidence interval, CV covariate, eGFR estimated glomerular filtration rate, IIV interindividual variability, V volume of distribution, SE standard error, Q intercompartimental clearance
